# Supplementary figures and images for: Genomic and enzymatic insights into α-amylase-producing Bacillus spizizenii strains isolated from Isfahan province, Iran
Source: PLoS One. 2025 Dec 18;20(12):e0333668. doi: 10.1371/journal.pone.0333668 (PMC12714195; doi:10.1371/journal.pone.0333668)

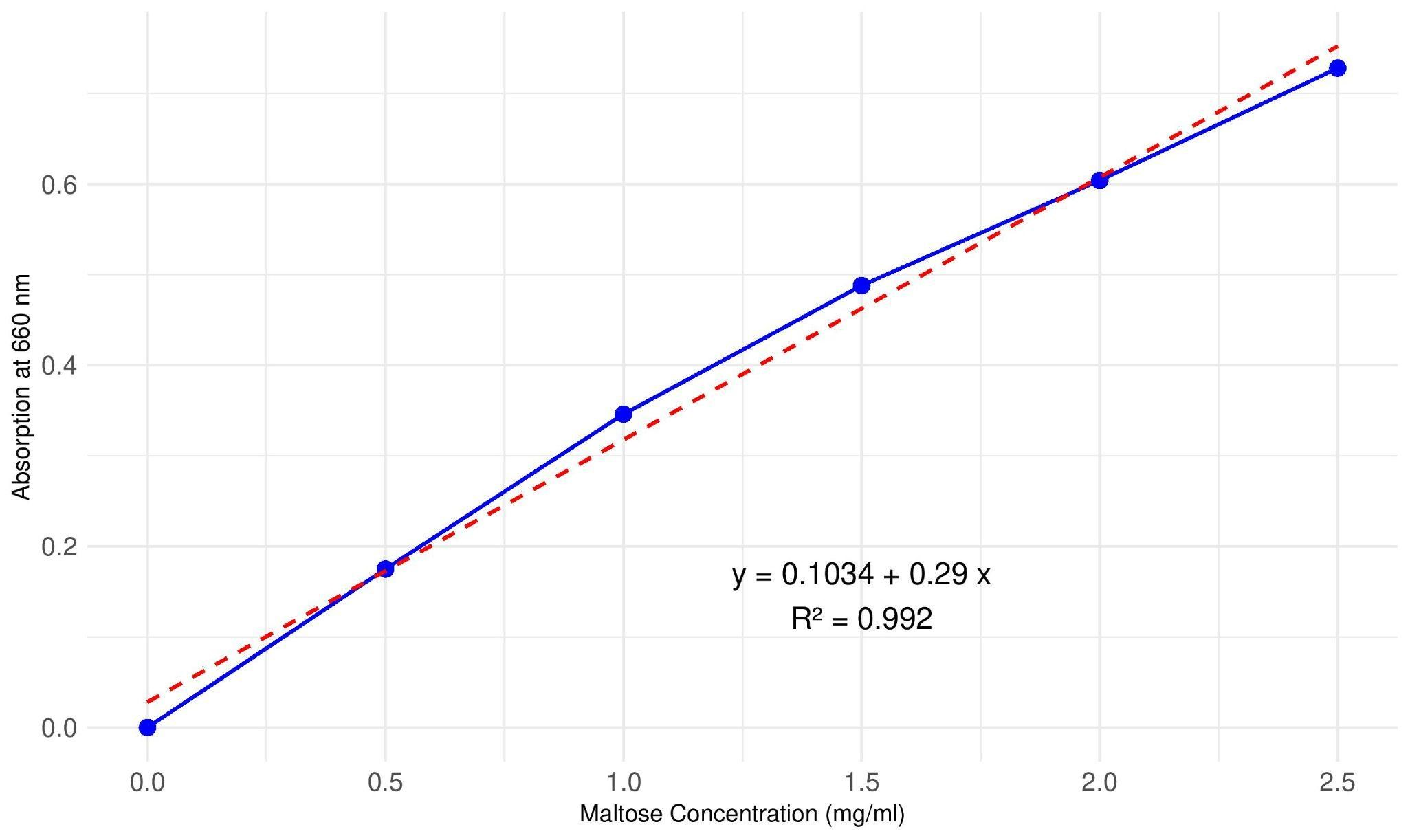

Supplement: S1 Fig — (TIFF) [file pone.0333668.s001.tiff]

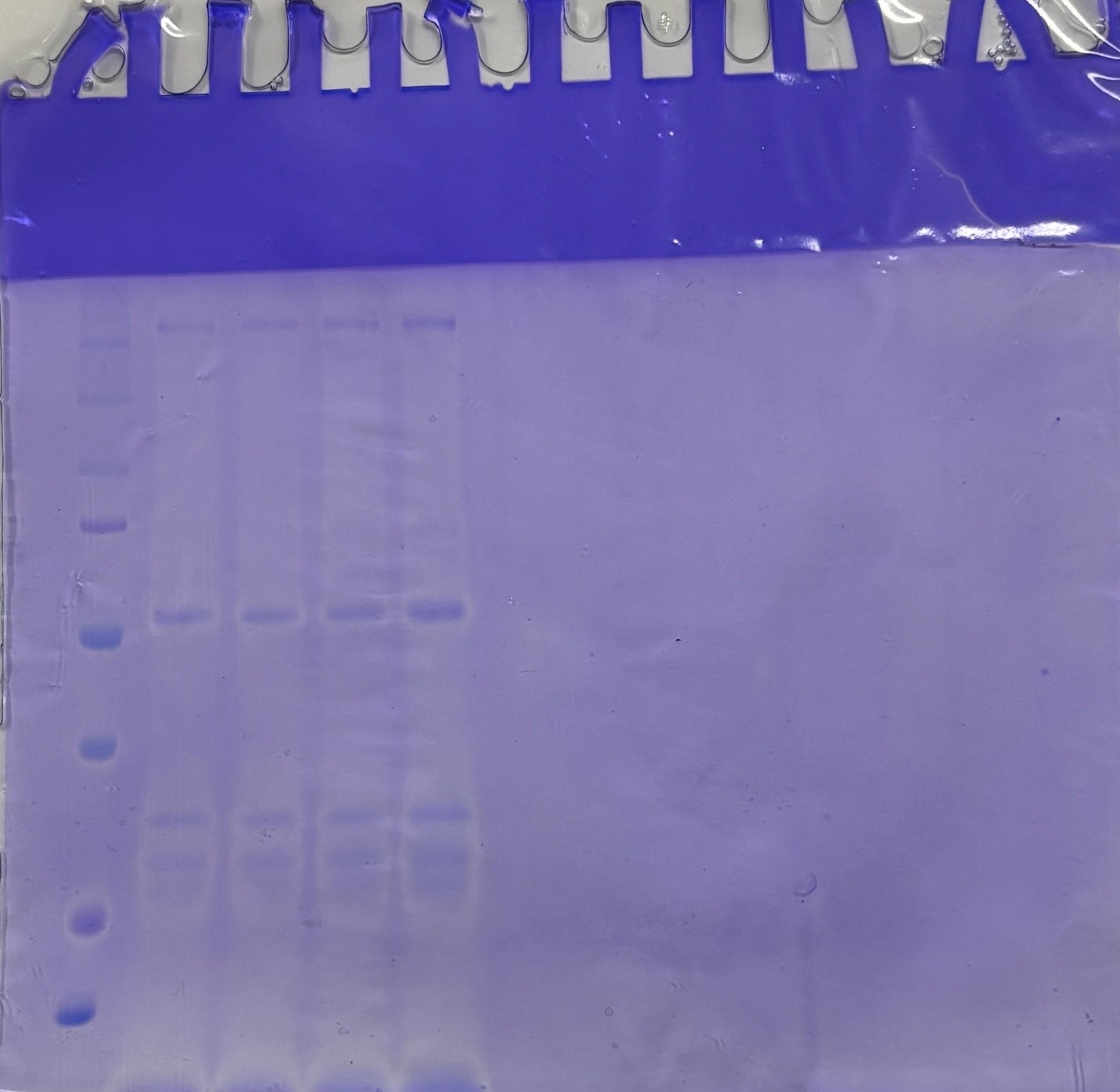

Supplement: S2 Fig — The complete gel image shows the analysis of partially purified α-amylase extracts. Lanes are as follows: Lane 1, protein molecular weight marker; Lane 2, ammonium sulfate precipitate from strain S1; Lane 3, precipitate from strain S3; Lane 4, precipitate from strain S4; Lane 5, precipitate from strain S5. The arrow highlights the position of the ~ 62 kDa α-amylase band. (TIFF) [file pone.0333668.s002.tiff]
