# Supplementary material for: Genomic and enzymatic insights into α-amylase-producing Bacillus spizizenii strains isolated from Isfahan province, Iran
Source: PLoS One. 2025 Dec 18;20(12):e0333668. doi: 10.1371/journal.pone.0333668 (PMC12714195; doi:10.1371/journal.pone.0333668)
Supplement: S1 File — Two-factor ANOVA results examining the effects of strain and pH over time at different Tm. (PDF) [file pone.0333668.s003.pdf]

Table S1: Two-factor ANOVA results examining the effects of strain and pH over time at 30 °C.

Source of Variation	df	Mean Square	F	Pr > F
Strain	4	16046847308	24482.4	< 0.0001
pH	4	751859802	1147.10	< 0.0001
Strain × pH	16	35333291	53.91	< 0.0001
Error	50	6655443.6		

In the table, **, *, ns indicate no significance, significance at the 0.05 and 0.01 probability levels, respectively.

Table S2: Two-factor ANOVA results examining the effects of strain and pH over time at 40 °C.

Source of Variation	df	Mean Square	F	Pr > F
Strain	4	16259179407	45117.6	< 0.0001
pH	4	688488900	1910.49	< 0.0001
Strain × pH	16	29525741	81.93	< 0.0001
Error	50	360373.40		

In the table, **, *, ns indicate no significance, significance at the 0.05 and 0.01 probability levels, respectively.

Table S3: Two-factor ANOVA results examining the effects of strain and pH over time at 50 °C.

Source of Variation	df	Mean Square	F	Pr > F
Strain	4	14348663154	57893.3	< 0.0001
pH	4	950301815	3834.23	< 0.0001
Strain × pH	16	44507251	179.58	< 0.0001
Error	50	247847		

In the table, **, *, ns indicate no significance, significance at the 0.05 and 0.01 probability levels, respectively.

Table S4: Two-factor ANOVA results examining the effects of strain and pH over time at 60 °C.

Source of Variation	df	Mean Square	F	Pr > F
Strain	4	12351757738	31477.5	< 0.0001
pH	4	893745540	2277.64	< 0.0001
Strain × pH	16	67333684	171.59	< 0.0001
Error	50	392399		

In the table, **, *, ns indicate no significance, significance at the 0.05 and 0.01 probability levels, respectively.

Table S5: Two-factor ANOVA results examining the effects of strain and pH over time at 70 °C.

Source of Variation	df	Mean Square	F	Pr > F
Strain	4	12543171388	13780.4	< 0.0001
pH	4	891554461	979.50	< 0.0001
Strain × pH	16	96907124	106.47	< 0.0001
Error	50	45510824		

In the table, **, *, ns indicate no significance, significance at the 0.05 and 0.01 probability levels, respectively.

Table S6: Two-factor ANOVA results examining the effects of strain and pH over time at 80 °C.

Source of Variation	df	Mean Square	F	Pr > F
Strain	4	9553800977	13707.0	< 0.0001
pH	4	732130184	1050.40	< 0.0001
Strain × pH	16	185445124	266.06	< 0.0001
Error	50	697001.20		

In the table, **, *, ns indicate no significance, significance at the 0.05 and 0.01 probability levels, respectively.

Table S7: Two-factor ANOVA results examining the effects of strain and pH over time at 90 °C.

Source of Variation	df	Mean Square	F	Pr > F

Strain	4	8108271752	19215.8	< 0.0001
pH	4	466639592	1105.89	< 0.0001
Strain × pH	16	167404153	396.73	< 0.0001
Error	50	421958		

In the table, **, *, ns indicate no significance, significance at the 0.05 and 0.01 probability levels, respectively.
