# Supplementary material for: Genomic and enzymatic insights into α-amylase-producing Bacillus spizizenii strains isolated from Isfahan province, Iran
Source: PLoS One. 2025 Dec 18;20(12):e0333668. doi: 10.1371/journal.pone.0333668 (PMC12714195; doi:10.1371/journal.pone.0333668)
Supplement: Attachment — Submitted filename: Response to Reviewers.docx [file pone.0333668.s005.docx]

**Dear Editor and Reviewers,**

I hope this message finds you well. We truly appreciate the time and effort you invested in thoroughly assessing our work. Your insightful comments and constructive criticisms have immensely contributed to improving the quality and clarity of our research. I want to assure you that we have carefully reviewed each of the comments provided by the reviewers and have made revisions to address concerns and suggestions. Below, we outline how we have incorporated their feedback into the revised manuscript:

***Editor comments#:***

***Comment 1:*** *Please upload a Response to Reviewers letter which should include a point by point response to each of the points made by the Editor and / or Reviewers. (This should be uploaded as a 'Response to Reviewers' file type.) Please follow this link for more information: http://blogs.PLOS.org/everyone/2011/05/10/how-to-submit-your-revised-manuscript.*

**Response:** Thank you for the reminder. We have now uploaded a detailed 'Response to Reviewers' document that includes a point-by-point response to all comments.

***Comment 2:*** *In your Methods section, please provide additional information regarding the permits you obtained for the work. Please ensure you have included the full name of the authority that approved the field site access and, if no permits were required, a brief statement explaining why.*

**Response:** No specific permits were required for this study as it did not involve endangered or protected species. We have added a brief statement to the Methods section to clarify this.

***Comment 3:*** *PLOS ONE now requires that authors provide the original uncropped and unadjusted images underlying all blot or gel results reported in a submission’s figures or Supporting Information files. This policy and the journal’s other requirements for blot/gel reporting and figure preparation are described in detail at https://journals.plos.org/plosone/s/figures#loc-blot-and-gel-reporting-requirements and https://journals.plos.org/plosone/s/figures#loc-preparing-figures-from-image-files. When you submit your revised manuscript, please ensure that your figures adhere fully to these guidelines and provide the original underlying images for all blot or gel data reported in your submission. See the following link for instructions on providing the original image data:* [*https://journals.plos.org/plosone/s/figures#loc-original-images-for-blots-and-gels*](https://journals.plos.org/plosone/s/figures#loc-original-images-for-blots-and-gels)*. In your cover letter, please note whether your blot/gel image data are in Supporting Information or posted at a public data repository, provide the repository URL if relevant, and provide specific details as to which raw blot/gel images, if any, are not available. Email us at plosone@plos.org if you have any questions.*

**Response:** The original uncropped gel images are now included in the Supporting Information files, and we have adhered to all blot/gel reporting requirements as specified.

***Comment*** *5: Please note that funding information should not appear in any section or other areas of your manuscript. We will only publish funding information present in the Funding Statement section of the online submission form. Please remove any funding-related text from the manuscript.*

**Response:** We have removed all funding-related text from the manuscript body as requested.

***Additional Editor Comments#:***

*Dear respected Authors,*

*I would like to inform you that, based on the feedback received from the reviewers, I have concluded that your manuscript requires a major revision. Please review all the comments provided by the reviewers and adjust your manuscript accordingly.*

***Comment 1****: Regarding your title, please specify the exact region of bacterial isolation in your title.*

**Response:** We thank the editor for this valuable suggestion. Since our study focused on two main strains (S1 and S3), we have revised the title to include the provinces of isolation. The updated title now reads:

**“Genomic and enzymatic insights into α-amylase-producing *B. spizizenii* strains isolated from Isfahan Province, Iran”**

***Comment 2****: In your introduction section indicate that Bacillus spizizenii (formerly classified under B.subtilis group).*

**Response:** We appreciate the editor’s insightful comment. As suggested, we have revised the Introduction to indicate the taxonomic status of *B. spizizenii*.

***Editor comments#:***

***Comment 1****: While you have isolated 60 bacterial strains, you have not provided detailed information regarding their identities. It would be helpful to include specific names or classifications of these bacteria, along with any relevant context, such as their sources or ecological significance.*

**Response:** We thank the reviewer for this insightful comment. As suggested, we have now revised Table 1 to include the specific strain designations (e.g., S1, S3, S4, S5) for the samples from which the most promising isolates were obtained.

***Comment 2****: For your polyphasic taxonomy studies, it is essential to incorporate both morphological and biochemical tests for the identification of Bacillus spp. Please specify the particular morphological characteristics observed (e.g., colony morphology, cell shape, and Gram staining results) as well as the biochemical tests conducted (e.g., catalase test, glucose fermentation).*

**Response:** We thank the reviewer for this suggestion. We have now provided detailed morphological characteristics, including colony morphology and Gram staining results, which confirm that all selected isolates are Gram-positive rods. Regarding biochemical tests, while we acknowledge their value, our study relied on a robust genomic polyphasic approach for definitive species-level identification. This included phylogenetic analysis of 16S/rpoB genes, whole-genome sequencing, and digital DNA-DNA hybridization (dDDH) and average nucleotide identity (ANI) calculations, which conclusively identified our strains as Bacillus spizizenii. Genomic methods provide higher resolution for distinguishing closely related Bacillus species than traditional biochemical profiling, and we are confident this approach fulfills the essential criteria for accurate taxonomic classification.

***Comment 3****: Please clarify where the whole genome sequencing protocol was performed. Include the facility or institution where this sequencing took place, along with any relevant details about the technology or platform used (e.g., Illumina, PacBio).*

**Response:** We thank the reviewer for this comment. As noted in the Genome sequencing, assembly, and annotation section, we have specified that whole-genome sequencing libraries were prepared and sequenced by **Novogene (UK)** using the **Illumina NovaSeq X Plus** platform. This description provides both the facility/company and the sequencing technology.

***Comment 4****: In your phylogenetic dendrogram, provide a clear description of the method you employed for constructing it (e.g., maximum likelihood, neighbor-joining). Additionally, include information on the bootstrap values used to assess the reliability of the tree's branches.*

**Response:** We thank the editor for this valuable suggestion. The requested information has now been included in the descriptions for Figures 3 and 4.

***Comment 5****: It is important to include a standard strain as a reference point in your identification process. This should be clearly stated, and each test used for identification should be organized into a table format for clarity and ease of comparison.*

**Response:** We thank the reviewer for this constructive feedback. We have now clearly stated in the “Morphological and molecular characterization” and “Genome sequencing” sections that Bacillus spizizenii TU-B-10T (DSM 15031) was used as the primary reference strain for phylogenetic analysis and genomic comparisons (dDDH and ANI).

***Comment 6****: Incorporate the purification image from the supporting information directly into the body of your manuscript.*

**Response:** We thank the reviewer for this valuable suggestion. As recommended, we have now incorporated the SDS-PAGE image demonstrating the purification of the α-amylase enzyme directly into the main body of the manuscript.

***Comment 7****: Carefully review your reference section and ensure that all Persian entries are removed for clarity and coherence.*

**Response:** We thank the reviewer for this important correction. We have carefully reviewed the entire reference list and have removed the entries that contained Persian script.

Reviewer Comments:

***Reviewer #1:***

*The manuscript presents a well-structured and clearly written study with a sound experimental design. The authors have successfully combined microbiological isolation, enzymatic characterization, phylogenetic analysis, and in silico modeling to investigate alpha-amylase-producing Bacillus strains. The objectives are well-defined, the methods are generally appropriate, and the overall flow of the manuscript supports comprehension. However, a number of clarifications, methodological details, and data inconsistencies need to be addressed to improve the manuscript’s scientific rigor, reproducibility, and alignment with standard academic practices.*

***Comment 1****: Confirmation of Vegetative Cell Contribution to Enzyme Production: The study identifies four Bacillus strains, which are capable of spore formation. It is unclear how the authors confirmed that the observed enzyme stability across a broad pH range and temperatures is attributed to vegetative cells rather than spores. Since the enzyme was extracted from culture supernatants, it is possible that the secreted enzymes were produced before sporulation. Clarification on the timing of enzyme harvest relative to the bacterial growth phase is recommended.*

**Response:** Thank you for this insightful comment regarding the potential contribution of spores to the observed enzyme stability. We confirm that the reported thermostability and pH tolerance are intrinsic properties of the secreted enzyme, not an artifact of sporulation, for the following reasons:

- Timing of Enzyme Harvest: As detailed in our Results section (Growth pattern and amylase activity), enzyme activity was harvested at the point of peak production, which occurred at 24 hours for all high-producing strains (S1, S3, S4, S5). Microscopic examination (Gram stain) of cultures at this 24-hour time point confirmed the overwhelming presence of vegetative cells with no observable spores, indicating that harvesting occurred during the late logarithmic/early stationary phase, prior to the onset of significant sporulation.
- Source of the Enzyme: The enzyme activity was measured from the cell-free supernatant after centrifugation. This protocol is specifically designed to isolate only extracellular, secreted enzymes. Any intracellular enzymes or enzymes associated with spores would have been pelleted during centrifugation and discarded.
- Nature of the Measurement: The stability assays were performed on this cell-free, supernatant-derived enzyme preparation. Therefore, the stability profiles we report reflect the inherent properties of the soluble, secreted enzyme in solution, independent of any cellular structures.

We have now clarified the timing of the harvest (24h, pre-sporulation) and its rationale in the Methods section to prevent any future ambiguity.

***Comment 2****: Cultivation Temperature: The authors used a cultivation temperature of 37°C during the enzyme activity assay. Given that the strains were isolated from environmental sources, why was this temperature selected instead of 30°C, which is more typical for environmental Bacillus species?*

**Response:** Thank you for raising this important methodological consideration. We selected 37°C for cultivation based on three key factors:

- Preliminary Optimization: Screening tests showed 37°C maximized both growth and enzyme production compared to 30°C, likely due to enhanced metabolic activity.
- Literature Precedent: Many industrial *Bacillus* spp. (e.g., B. *licheniformis*, B. *amyloliquefaciens*) are routinely cultured at 37°C for enzyme production, as this balances yield and stability.

We added these ponits to the main text.

***Comment 3****: Negative Control for α-Amylase Activity: While the manuscript mentions the use of a negative control for α-amylase production, the nature of this control is not specified. Please provide details regarding the strain or condition used as the negative control.*

**Response:** We sincerely appreciate the reviewer’s attention to methodological detail. As suggested, we have now explicitly clarified the nature of the negative control in the revised manuscript. The negative control consisted of the reaction mixture (1% starch in buffer) without the enzyme, which confirmed the absence of non-enzymatic starch hydrolysis.

***Comment 4****: DNS Assay – Absorbance Wavelength: The DNS method is typically measured at 540 nm, but the authors report measurements at 660 nm. Please clarify whether this is a methodological adaptation or an error.*

**Response:** We sincerely thank the reviewer for catching this discrepancy. The mention of 660 nm in the Results section was indeed a typographical error - all measurements were properly conducted at 540 nm as correctly stated in our Methods section. We have now:

- Corrected all instances of "660 nm" to "540 nm" in the Results section and Figure 1
- Verified consistency between Methods and Results throughout the manuscript

We apologize for this oversight and appreciate the reviewer's careful attention to detail, which has helped improve the accuracy of our manuscript.

***Comment 5****: Dialysis Procedure: The molecular weight cutoff (MWCO) of the dialysis membrane used is not specified. This information is essential for reproducibility and should be included.*

**Response:** We thank the reviewer for this important technical suggestion. We have now added the molecular weight cutoff (MWCO) details of the dialysis membrane in the revised Methods section (subsection "Partial purification of the enzyme") as follows:

*"The concentrated enzyme solution was dialyzed against deionized water using a cellulose membrane with a 12-14 kDa MWCO (Sigma-Aldrich, #D9777) to remove residual ammonium sulfate."*

***Comment 6****: Centrifugation Speed Reporting: The manuscript reports centrifugation at 15,000 rpm, whereas elsewhere, centrifugal force is expressed in ×g. For consistency, all centrifugation speeds should be presented in relative centrifugal force (RCF, ×g). Additionally, labeling this step as “high-speed centrifugation” would improve clarity.*

**Response:** We sincerely apologize for this inconsistency and appreciate the reviewer’s valuable feedback. We have now revised the manuscript to convert all centrifugation speeds to relative centrifugal force (RCF, ×g) for consistency

***Comment 7****: Docking Study – Gene or Protein?: The text states that docking was used to “evaluate the interaction dynamics between the alpha-amylase gene and its respective ligand molecules.” Docking is typically performed at the protein level, not the gene level. Please clarify whether the docking analysis was conducted using the predicted 3D structure of the alpha-amylase protein, as expected.*

**Response:** We sincerely thank the reviewer for catching this important terminology error. The reviewer is absolutely correct - the docking study was indeed performed using the predicted 3D structure of the alpha-amylase protein (not the gene). We have carefully revised the manuscript

***Comment 8****: Missing Method Section: Growth Pattern and Enzyme Activity: The methods used to study growth pattern and amylase activity are described in the Results section but not in the Methods. To improve clarity and organization, this section should be moved or referenced properly within the Methods.*

**Response:** Thank you for this excellent suggestion to improve the manuscript's organization. We have revised the manuscript accordingly by adding the detailed methodological description of the growth pattern to the methods section, dedicated section in the Methods titled "Bacterial growth and enzyme activity assay

***Comment 9****: Selective Culture Medium Composition: The ingredients or selective agents used in the “selective culture medium” are not described. Please include full composition and purpose of the medium.*

**Response:** Thank you for highlighting this omission. We have now revised the Materials and Methods section to include the full composition and purpose of the selective culture medium

***Comment 10****: Initial Maltose Concentration: The initial maltose concentration in the culture medium is not reported, which is essential for evaluating substrate reduction and enzyme efficiency.*

**Response:** Thank you for this important observation. We have now clarified in the Methods section that the initial maltose concentration in the culture medium was 2.5% (w/v), as this concentration was optimized during preliminary screening for maximal α-amylase induction.

***Comment 11:*** *Discrepancy Between Text and Figure 2A: The text states that "strain S1 surpassed S3 in enzyme activity at 50°C and pH 7," but Figure 2A shows strain S3 having the highest activity at that point. Please resolve this inconsistency.*

**Response:** We thank the reviewer for identifying this discrepancy. We have revised the text to accurately reflect Figure 2A, confirming that strain S3 showed the highest activity at 50°C and pH 7 (32,465 U/g), and have verified consistency throughout the manuscript.

***Comment 12****: Inconsistency Between Figures 2A and 2B: The enzyme activity patterns in Figures 2A and 2B appear to differ. For example, S3 maintains high activity at 70°C in 2A, while the top activity at that temperature in 2B appears to belong to S1. Clarification or correction is needed.*

**Response:** Thank you for your careful observation regarding Figures 2A and 2B. We appreciate the opportunity to clarify this point. The figures represent two different datasets:

- Figure 2A presents enzyme activity as a function of strain and temperature, showing the performance of each strain across the tested temperatures.
- Figure 2B is a heatmap that presents enzyme activity as a function of strain and pH, showing the performance of each strain across the tested pH levels at each specific temperature.

The apparent discrepancy is not an error but a result of the different ways the complex, multi-dimensional data (Strain × Temperature × pH) are visualized. This means the activity values and patterns between the two figures are not directly comparable, as they were measured under different foundational conditions.

***Comment 13****: Missing rpoB Primer Sequences: The primer sequences used for amplifying the rpoB gene are not provided. These should be included for transparency and reproducibility.*

**Response:** We thank the reviewer for their careful reading. We recognize that both 16S and rpoB primer sequences were included in the Methods section.

***Comment 14****: Missing Reference or Sequences for 16S rRNA Primers: No reference or sequence information is given for the 16S rRNA primers. Please add this to the manuscript.*

**Response:** We thank the reviewer for pointing this out. We added references for both markers

***Comment 15****: Missing SDS-PAGE Results: Although protein extraction and purification are discussed, the SDS-PAGE results (e.g., molecular weight confirmation) are not presented. These are standard for enzyme characterization and should be included.*

**Response:** We appreciate the reviewer's important point. We confirm that the SDS-PAGE results showing protein purification and molecular weight validation were already included as Figure 6 the main body of manuscript.

***Comment 16****: No Presentation of α-Amylase Gene Sequence: The nucleotide or amino acid sequence of the alpha-amylase gene under investigation is not shown. Inclusion of this data would strengthen the study and allow for future comparison.*

**Response:** We thank the reviewer for this valuable suggestion. In response, we included the complete nucleotide sequence of the α-amylase gene in Supplementary file.

***Comment 17****: Missing GOR4 Analysis Data: While the GOR4 tool is mentioned for secondary structure prediction, the results of this analysis are not included. Please add the relevant data or images.*

**Response:** We thank the reviewer for their careful assessment. We confirm that the GOR4 secondary structure prediction results were included in the original manuscript (Results section: "Computational analysis"), which reported: "Secondary structure prediction indicated that the protein comprises 20.33% alpha helices (Hh), 24.13% extended strands (Ee), and 55.54% random coils (Cc)."

***Comment 18****: Missing 3D Structure Validation Data: The manuscript states that the 3D structure was validated, but does not provide Ramachandran plots, ERRAT, or Verify3D outputs. These are essential for validating structural models and should be included in the supplementary materials or main text.*

**Response:** We thank the reviewer for emphasizing the importance of structural validation. As requested, we have now included detailed validation metrics in the Results section (subsection "Molecular Docking"), reporting:

- ERRAT score: 95.4 (exceeds the >80 threshold for high-quality models)
- Verify3D: 93.8% residues with 3D-1D score ≥0.1 (well above the 80% acceptability threshold)

***Comment 19****: Missing Strain Location Information: The geographical origin or sampling location of strains S1, S3, S4, and S5 is not mentioned. Including this information would provide important ecological and contextual relevance to the study.*

**Response:** Thank you for this valuable suggestion. We have now added the precise geographical origins of the high-performing strains S1, S3, S4, and S5 to Table 1 in the manuscript.

***Reviewer #2:***

*Questions for the Corresponding Author*
***Comment 1****: You report the enzyme activity as high as 34,121 U/g. Can you provide the exact experimental setup, replicates, and error margins that support this claim? How was U/g converted from absorbance?*

**Response:** Thank you for your question regarding enzyme activity calculations. We have now added detailed methodological clarification in the Methods section, explaining that activity (U/g) was calculated through a two-step process: (1) U/mL was determined from μmol maltose produced (via standard curve) divided by reaction time and enzyme volume, then (2) normalized to biomass by dividing U/mL by mg biomass per mL enzyme solution and multiplying by 1000.

***Comment 2****: Why was there no use of enzyme kinetics (Km, Vmax) or substrate specificity testing to confirm functional superiority over existing α-amylases?*

**Response:** Thank you for this valuable suggestion. While kinetic parameters (Km, Vmax) would provide deeper functional insights, our study focused on establishing the biotechnological potential of these *B. spizizenii* strains through: (1) high-yield enzyme production (34,121 U/g), (2) broad pH/thermal stability (pH 4-9, 30-80°C), and (3) genomic sequence and characterization, all critical industrial screening criteria. We emphasize that these robust activity/stability metrics under application-relevant conditions strongly indicate functional superiority, even without kinetic profiling. However, we fully acknowledge the importance of kinetic characterization and have now explicitly highlighted this limitation while proposing such analyses for future research.

***Comment 3****: What mechanistic evidence supports the claimed thermostability and pH stability of the enzyme beyond in vitro activity measurements (e.g., structural analysis or melting temperature assays)?*
**Response:** Thank you for this insightful question regarding the mechanistic basis for the enzyme’s stability. While our study primarily focused on in vitro functional characterization (activity under stress conditions), we agree that deeper structural insights would further strengthen our claims. The following evidence supports the observed stability:

- In Silico Structural Analysis: The predicted structure (validated by Ramachandran plot, ERRAT, and Verify3D) showed a high proportion of random coils (55.54%), which may contribute to structural flexibility and resilience under thermal stress.
- Genetic Basis: The enzyme belongs to the GH13 family, which is known for its robust α-amylases. Sequence analysis showed 93.6% similarity to the well-characterized, thermostable AmyE from B. subtilis, implying conserved stability mechanisms.
- Experimental Design: Our activity assays across a broad range of temperatures (30–90°C) and pH (3–11) explicitly tested stability, not just optimal activity. The enzyme retained significant function after incubation at extreme conditions (e.g., >60% activity at 80°C), indicating inherent robustness.

We acknowledge that techniques like circular dichroism (for melting temperature) or X-ray crystallography would provide direct mechanistic evidence and have now explicitly stated this as a focus for future research in the Conclusion section.

***Comment 4****: Did you validate the AlphaFold2-predicted structure experimentally (e.g., CD spectroscopy, crystallography)? How do you confirm that the docking simulations are biologically meaningful?*

**Response:** Thank you for this important question. While we did not perform experimental validation via CD spectroscopy or crystallography due to the significant time and cost constraints associated with these techniques, we rigorously validated the AlphaFold2-predicted structure using computational approaches: ERRAT analysis yielded a 95.4% quality score, and Verify3D confirmed 93.8% of residues had compatible 3D-1D scores (≥0.1). For docking studies, we ensured biological relevance by focusing on conserved catalytic residues and demonstrating strong binding affinities (-8.4 kcal/mol with maltotetraose) consistent with known GH13-family amylase interactions. While experimental validation would be valuable, these computational methods provide robust preliminary validation and align with current practices for in silico studies, particularly for novel enzyme characterization in early-stage research. We acknowledge that experimental validation would further strengthen these findings and propose this as future work if the enzyme advances toward industrial applications.

***Comment 5****: Your docking analysis shows -8.4 kcal/mol affinity for maltotetraose. Did you perform validation using known inhibitors or competitive ligands to assess docking reliability?*

**Response:** Thank you for this excellent methodological question. To ensure the reliability of our docking results, we performed the following validation steps:

- Internal Validation via Redocking: The predicted binding pose of maltotetraose was compared to crystallographic data of highly homologous B. subtilis AmyE (PDB: 1BAG) complexed with maltopentaose. The root-mean-square deviation (RMSD) of the ligand pose was <2.0 Å, confirming the accuracy of our docking protocol and parameters.
- Competitive Docking with Known Inhibitor: We docked acarbose, a known potent inhibitor of GH13 α-amylases, into our model. The resulting binding affinity (-10.2 kcal/mol) and binding pose aligned closely with crystallographic data from Bacillus-acarbose complexes (e.g., PDB: 1FDK), with key interactions conserved in the active site.

These results confirm that our docking methodology reliably reproduces known experimental interactions and provides confidence in the novel maltotetraose binding data. We have now added a summary of this validation to the Methods section under "Molecular Docking".

***Comment 6****: Given that B. spizizenii was previously classified under B. subtilis, what molecular markers (e.g., ANI, dDDH, or pan-genome features) unequivocally separate your isolates from known strains?*
**Response:** Thank you for raising this important taxonomic consideration. As detailed in our "Genome Sequencing" section, we employed ANI (Average Nucleotide Identity) and dDDH (digital DNA-DNA Hybridization) to conclusively differentiate our isolates from closely related *Bacillus* strains. Our results show:

- ANI values of 99.02-99.09% between our strains (*S1/S3*) and B. spizizenii (exceeding the 95-96% species threshold), but only 93.6% to B. subtilis strain 168 (P00691), confirming closer alignment with B. spizizenii.
- dDDH estimates of 93.1% (GLM method) with B. spizizenii, far above the 70% species cutoff, and <70% with B. subtilis.

***Comment 7****: Why was 16S rRNA used for taxonomic classification despite its known limitations in distinguishing Bacillus species? Why not employ multi-locus sequence analysis (MLSA)?*
**Response:** We thank the reviewer for this comment. As addressed in response to *Comment #15*, we have now included a whole-genome-based phylogenetic tree (TYGS analysis) to resolve taxonomic relationships with higher resolution.

***Comment 8****: What genomic features or genes in the B. spizizenii strains contribute to the thermal and pH tolerance beyond the α-amylase gene itself?*
**Response:** Thank you for this insightful question regarding the genomic basis of the observed stability. While the α-amylase gene itself encodes a robust enzyme, the thermal and pH tolerance of the B. spizizenii strains is likely a multifactorial trait supported by several genomic features identified in our whole-genome analysis:

- Osmoregulation and pH Homeostasis: We identified key genes involved in synthesizing and transporting compatible solutes (e.g., proline, glycine betaine), which protect cellular machinery from osmotic and thermal stress. Genes for electrogenic Na⁺/H⁺ antiporters (e.g., nhaC) and ATPases were also present, which are critical for maintaining intracellular pH across a broad external range.
- Cell Envelope Modifications: The genomes contain genes for the synthesis of branched-chain fatty acids, which increase membrane fluidity and integrity at high temperatures. Additionally, genes associated with teichoic acid and peptidoglycan biosynthesis were identified, which can contribute to cell wall stability under alkaline or acidic conditions.
- Antioxidant Defense: Genes for catalase (katA), superoxide dismutase (sodA), and thioredoxin systems were present, which mitigate oxidative damage often exacerbated by thermal stress.

We have now added a brief result of these supporting genomic features to the result section to provide a more comprehensive explanation for the strains' robust phenotypes.

***Comment 8****: Was the gene copy number of α-amylase determined in the genome? Could gene dosage explain the high production rate?*

**Response:** Thank you for this insightful question regarding the genetic basis of the high enzyme production. We did not determine the copy number of the α-amylase gene in this study, as our genomic characterization focused on identifying the gene presence, sequence, and structural features rather than quantification. While gene dosage is a plausible factor for high production rates, we note that the α-amylase gene in both S1 and S3 strains appears to be present as a single copy in the assembled genomes, based on read depth and annotation consistency.

***Comment 9****: How do you address potential codon bias or expression bottlenecks if the gene were to be heterologously expressed in a commercial host?*

**Response:** We thank the reviewer for raising the possibility of codon bias and downstream expression bottlenecks. As outlined in our response to Comment 21 and now reported in the Results, codon adaptation index (CAI) analysis indicates high compatibility of the α-amylase ORF with Bacillus subtilis (CAI = 0.81), and moderate compatibility with E. coli (CAI = 0.64) and Pichia pastoris (CAI = 0.65).

***Comment 10****: Was there any testing for protease resistance, a critical property for feed industry applications, especially in the digestive tract environment?*

**Response:** We appreciate this important point. Protease-resistance testing was not performed in the present study. Our work was scoped to isolate/identify the α-amylase–producing strains, characterize enzyme activity and stability across pH/temperature, and genome sequencing. Robust evaluation of feed-relevant protease tolerance (e.g., survival in simulated gastric/intestinal environments) requires a distinct experimental design, including controlled assays with pepsin/trypsin/chymotrypsin or pancreatin under physiologically relevant pH, ionic strength, and residence times, ideally in the presence of feed matrices. Implementing these assays would also necessitate additional controls (commercial benchmarks), optimized readouts (residual activity/half-life), and, potentially, formulation variables (e.g., Ca²⁺ supplementation or microencapsulation), which were beyond the scope and resources of the current work.

***Comment 11****: How reproducible were the enzyme activities across biological replicates? Did you test enzyme production in different batches or fermentation conditions?*

**Response:** Thank you for this important question regarding the reproducibility of our results. We are pleased to confirm that the enzyme activities were highly reproducible across replicates. All reported enzyme activity assays were performed with triplicate biological replicates (n=3 independent cultures), demonstrating high reproducibility. Full statistical details are provided in Supplementary Tables.

***Comment 12****: You mention SDS-PAGE analysis. Was the band at ~62 kDa verified by mass spectrometry or western blot to confirm its identity as α-amylase?*

**Response:** We sincerely thank the reviewer for this valuable suggestion. In the current study, the ~62 kDa band observed on SDS-PAGE was identified based on the predicted molecular weight of the α-amylase enzyme and its strong correlation with the measured enzymatic activity profile from corresponding gel fractions. However, mass spectrometry or western blot validation was not performed, as this would require specialized facilities and resources beyond the scope of our present work.

***Comment 13****: How were protein concentrations normalized across strains when comparing enzyme activity? Was Bradford or BCA assay used?*

**Response:** Thank you for this critical methodological question. We confirm that enzyme activity was normalized using a standardized and rigorous approach:

For the reported enzyme activity comparisons (U/g), normalization was based on biomass concentration (dry cell weight), not direct protein concentration in the crude extract. Specifically:

- Cell Harvesting: Cultures were centrifuged, and the cell pellets were washed and lyophilized to determine the dry weight (g/L of culture).
- Activity Calculation: Enzyme activity in the cell-free supernatant was then expressed as units per gram of dry cell weight (U/g DCW). This method directly measures each strain's efficiency in producing the enzyme per unit of biomass, which is a standard metric for comparing microbial productivity.

We selected this approach because it avoids potential inaccuracies from crude extract protein assays (e.g., Bradford/BCA), which can be influenced by varying levels of other secreted proteins across strains

***Comment 14****: You suggest these enzymes are suitable for animal feed processing. Did you test enzyme activity under simulated gastric conditions (e.g., pH 2 + pepsin)?*
**Response:** Thank you for this highly relevant suggestion regarding animal feed application. We agree that testing under simulated gastric conditions (e.g., low pH with pepsin) is a critical step for validating an enzyme's suitability for animal feed. However, such stability assays require highly purified enzyme and controlled in vitro digestion models, which were beyond the scope of this initial discovery and characterization study. Our primary focus was on identifying novel microbial strains with high enzyme production and robust activity under a broad range of general industrial conditions (pH, temperature).

***Comment 15****: Your phylogenetic tree is based on 16S and rpoB sequences. Why not include whole-genome-based phylogeny for higher resolution?*

**Response:** We appreciate the reviewer’s insightful suggestion regarding the use of whole-genome phylogeny for higher resolution. In this study, we initially employed *16S* rRNA and *rpoB* gene sequences to provide a broad phylogenetic framework, as these markers are widely used for taxonomic placement and allow direct comparison with historical datasets. However, we fully agree that whole-genome-based analyses offer superior resolution for delineating closely related strains. To address this, we have now conducted an additional phylogenomic analysis using the Type (Strain) Genome Server (TYGS) platform, which leverages whole-genome sequences to infer robust evolutionary relationships. The updated tree (Figure 4) confirms the close affiliation of strains S1 and S3 with *Bacillus spizizenii* TU-B-10, as suggested by the gene tree, but with significantly higher bootstrap support. Notably, the genome-based tree resolves finer-scale relationships, such as the clear sister-group pairing of S1 and S3, that were unresolved in the *16S*/*rpoB* phylogeny.

***Comment 16****: Were mobile genetic elements or plasmids present in the genomes that might encode the α-amylase or other industrially useful traits?*

**Response:** Thank you for this insightful question regarding the genomic context of the α-amylase gene. We did not specifically investigate mobile genetic elements (MGEs) or plasmids in this study, as our annotation and analysis focused primarily on chromosomal genes and pathways directly relevant to α-amylase function and stress tolerance. However, our whole-genome sequencing data did not detect any plasmids in the assembled genomes of strains S1 and S3. The α-amylase gene itself was located within the chromosomal scaffold in both strains.

***Comment 17****: You performed docking with maltotriose and maltotetraose. Why not validate these findings using enzyme-substrate kinetics to correlate in silico and in vitro results?*

**Response:** Thank you for your insightful suggestion regarding experimental validation of the docking results. While enzyme-substrate kinetics would indeed provide valuable complementary data, we focused on in silico approaches in this study due to three primary constraints:

- Research Scope: This work prioritized discovery and characterization of strains and α-amylase producers, with computational analysis and docking serving as a preliminary screen for substrate interactions to guide future functional studies.
- Resource Limitations: Kinetic assays (e.g., Michaelis-Menten) require purified enzyme in large quantities, optimized reaction conditions, and specialized equipment (e.g., stopped-flow systems), which were beyond the current project’s budget and timeline.
- Strategic Alignment: Our goal was to establish genetic potential and structural plausibility of the enzyme’s activity (via docking with catalytic residues and GH13-family conservation), which aligns with similar studies in early-stage enzyme characterization.
- Future Work: We fully acknowledge the importance of kinetic validation and have now explicitly proposed this as a next step in the Discussion section.

"While docking simulations identified strong interactions with ligands such as maltooligosaccharides, further studies measuring kinetic parameters (Km, kcat) are needed to quantify catalytic efficiency. Such experiments will be prioritized upon scaling up enzyme production for industrial applications."

***Comment 18****: In the enzyme purification step, what was the specific activity at each stage (crude, ammonium sulfate, dialysis)?*

**Response:** Thank you for this important technical question. While we focused on the functional characterization of the partially purified enzyme, we recognize that reporting stage-specific activity would provide valuable insight into the purification efficiency. Due to the preliminary nature of this study and our focus on identifying enzyme producers (rather than optimizing purification), we prioritized activity measurements only after dialysis for the following reasons:

- Methodological Focus: Our primary goal was comparative analysis of enzyme activity between strains under standardized conditions (post-dialysis), not purification yield optimization.
- Consistency: Measuring activity only after dialysis eliminated variability from salt inhibition effects that could skew comparisons between strains.

***Comment 19****: How do you account for the discrepancy in molecular weights of your α-amylase (~62 kDa) and commonly reported α-amylases from Bacillus species (typically ~55 kDa)?*

**Response:** Thank you for your observation regarding the molecular weight of our α-amylase. As discussed in the manuscript (Discussion section), the 62 kDa size of our *B. spizizenii* α-amylase falls within the well-documented range for *Bacillus* species (22–93 kDa), closely matching several validated homologs (e.g., *B. atrophaeus* AmyI at 61 kDa and *B. subtilis* KIBGE HAS at 58 kDa). This variation reflects natural diversity in domain architecture and potential post-translational modifications, with smaller (42–55 kDa) and larger (68–93 kDa) forms equally reported in literatures. Our enzyme’s functional performance (activity, stability) confirms its biological relevance despite this molecular weight difference, which we now emphasize more clearly in the revised discussion.

***Comment 20****: You reference strong ANOVA significance; was the data distribution checked for normality and homoscedasticity before applying ANOVA?*

**Response:** Thank you for your important methodological question. We confirm that all ANOVA assumptions were properly validated: normality was confirmed using Shapiro-Wilk tests (p > 0.05 for all groups) and Q-Q plot inspection, while homoscedasticity was verified via Levene's test (p > 0.05). To address multiple testing concerns, we applied Holm-Bonferroni correction to all post-hoc comparisons. These methodological details have now been explicitly added to the Methods section, and the Results section now notes the validation of these assumptions.

***Comment 21****: Have you performed codon usage analysis for the α-amylase gene, and how compatible is it with common expression systems like E. coli, Pichia, or Bacillus subtilis?*

**Response:** We sincerely appreciate this insightful suggestion. As now reported in the Results section, codon adaptation index (CAI) analysis revealed that our α-amylase gene exhibits:

- High compatibility with *B. subtilis* (CAI = 0.81), supporting its native expression efficiency.
- Moderate compatibility with *E. col*i (CAI = 0.64) and *Pichia pastoris* (CAI = 0.65), suggesting that rare codon optimization (e.g., for Arg/Lys) would be beneficial for these systems.
