# Supplementary material for: Genomic and enzymatic insights into α-amylase-producing Bacillus spizizenii strains isolated from Isfahan province, Iran
Source: PLoS One. 2025 Dec 18;20(12):e0333668. doi: 10.1371/journal.pone.0333668 (PMC12714195; doi:10.1371/journal.pone.0333668)
Supplement: Attachment — Submitted filename: Response_to_Reviewers_auresp_2.docx [file pone.0333668.s006.docx]

**Dear Editor and Reviewers,**

I hope this message finds you well. We truly appreciate the time and effort you invested in thoroughly assessing our work. Your insightful comments and constructive criticisms have immensely contributed to improving the quality and clarity of our research. I want to assure you that we have carefully reviewed each of the comments provided by the reviewers and have made revisions to address concerns and suggestions. Below, we outline how we have incorporated their feedback into the revised manuscript:

***Additional Editor Comments#:***

***Comment 1****: In my initial feedback, I requested that you specify the exact location of your isolation in the title. Thank you for adding "Isfahan province" in your revised version. I have noticed that you also screened samples from other provinces. Therefore, please clarify that you tested environmental samples from various provinces across Iran and that the best-performing strains were those isolated from Isfahan province (please include their specific code numbers). This information should be added to both the abstract and the materials and methods sections.*

**Response:** We thank the editor for this crucial point. We have made the following changes to the manuscript:

- Abstract: We have modified the text to specify that samples were collected from "various provinces of Iran, including Golestan, Mazandaran, Gilan, Kurdistan, and Isfahan." Furthermore, we now explicitly stated that the top strains S1 and S3 were "isolated from the Kuhrang water source and Gavkhouni Wetland in Isfahan province, respectively."
- Materials and Methods: To further improve clarity, we have also added a sentence at the beginning of the 'Effect of temperature and pH on alpha-amylase activity' subsection to specify which strains were used in this experiment and their origins.

***Reviewer #1:***

*All comments have been addressed appropriately, except for the following two points:*

***Comment 1****: Please provide Gram-staining images (if available) to verify and demonstrate the presence or absence of vegetative cells under the different conditions applied in the enzymatic assay.*

**Response:** We thank the reviewer for this request for additional visual evidence to support our previous clarification. We agree that providing microscopic images would further strengthen our manuscript. Unfortunately, while Gram staining was performed qualitatively to confirm cell morphology and the absence of spores at the time of harvest (as stated in the Methods), systematic photomicrography was not conducted as a standard part of our protocol for every assay condition. Therefore, we do not have high-quality, publication-ready images readily available from the original experiments.

However, we have revised the manuscript to explicitly state in the 'Bacterial growth and enzyme activity assay' section of the Methods that which Microscopic examination (Gram stain) of cultures at the 24-hour harvest time point confirmed the exclusive presence of vegetative cells with no observable spores, ensuring enzyme extraction occurred prior to sporulation.

***Comment 2****: It is essential to include the results of the negative control and integrate them into both the statistical analysis and the graphical representations of enzyme activity. This will ensure that the potential effect of starch auto-degradation in the culture medium is appropriately accounted for and not overlooked.*

**Response:** We thank the reviewer for raising this critical methodological point. In response to this comment, we have now added the data for the negative control to Figure 1 (as seen in the revised manuscript). This negative control, which consisted of the reaction mixture (1% starch in buffer) without any enzyme, was included in all assay runs.

The negative control, by definition, contains no bacterial cells and therefore produces zero biomass. Consequently, the concept of enzymatic activity normalized to biomass (U/g) is not mathematically or scientifically defined for the negative control. Its purpose is to validate the assay itself (which it does, as shown in Fig. 1), not to represent a biological producer of enzyme that can be compared on a specific activity basis. Therefore, while the negative control is crucial for validating the assay methodology (and is now included in Fig. 1), its inclusion in figures depicting specific enzymatic activity (U/g) produced by bacterial strains would be misleading, as it has no biological component to normalize to.
